# Nosocomial acquisition of methicillin-resistant *Staphyloccocus aureus *(MRSA) and extended-spectrum beta-lactamase (ESBL) *Enterobacteriaceae *in hospitalised patients: a prospective multicenter study

**DOI:** 10.1186/1471-2334-12-74

**Published:** 2012-03-29

**Authors:** Giulia De Angelis, Giovanni Restuccia, Silvia Venturiello, Roberto Cauda, Surbhi Malhotra-Kumar, Herman Goossens, Jacques Schrenzel, Evelina Tacconelli

**Affiliations:** 1 Department of Infectious Diseases, Università Cattolica del Sacro Cuore, Largo F. Vito, 1 00168 Rome, Italy; 2 Department of Medical Microbiology, Vaccine & Infectious Disease Institute, Campus Drie Eiken, University of Antwerp, S3, Universiteitsplein 1, Wilrijk, Antwerp B-2610, Belgium; 3 Genomic Research and Bacteriology Laboratories, Service of Infectious Diseases, Geneva University Hospitals, Rue Gabrielle-Perret-Gentil 4, Geneva 14 CH-1211, Switzerland

**Keywords:** MRSA, ESBL, Antibiotic resistance, SATURN, Antibiotic use

## Abstract

**Background:**

The risk of acquisition of antibiotic resistant-bacteria during or shortly after antibiotic therapy is still unclear and it is often confounded by scarce data on antibiotic usage.

Primary objective of the study is to compare rates of acquisition of methicillin-resistant *Staphylococcus aureus *and extended spectrum beta-lactamase-producing *Enterobacteriaceae *in hospitalised patients, after starting antibiotic therapy.

**Methods/Design:**

The study, running in three European hospitals, is a multicenter, prospective, longitudinal, observational cohort study funded from the European Community's Seventh Framework Programme [FP7/2007-2013] within the project 'Impact of Specific Antibiotic Therapies on the prevalence of hUman host ResistaNt bacteria' (acronym SATURN). Nasal and rectal screening for methicillin-resistant *Staphylococcus aureus *and extended spectrum beta-lactamase-producing *Enterobacteriaceae *will be obtained at hospital admission, discharge, at antibiotic start (t_0_, within one hour) and at the following intervals: day 3 (t_1_), 7 (t_2_), 15 (t_3_), and 30 (t_4_). Two nested case-control studies will be performed. The objective of the first study will be to define individual level of risk related to specific antibiotics. Patients acquiring methicillin-resistant *Staphylococcus aureus *and extended spectrum beta-lactamase-producing *Enterobacteriaceae *(cases) will be compared with patients not acquiring antibiotic-resistant strains after starting antibiotic therapy (controls; ratio 1:4). To define the impact of antibiotics on new acquisition of target antibiotic-resistant bacteria, a second nested case-control study will be done (ratio 1:4). Control group will be selected among patients not receiving antibiotics, admitted in the same ward on the day of the corresponding case, with negative cultures at admission. Epidemiological, clinical and microbiological data will be prospective collected.

**Discussion:**

The rationale of this study is to better understand the impact of antibiotic use on acquisition, selection and transmission of antimicrobial resistant-bacteria in European hospitals.

**Trial registration:**

ClinicalTrials.gov NCT01208519.

## Background

Despite many efforts, the control of nosocomial antibiotic-resistant infections is still a permanent and unresolved issue in healthcare institutions worldwide facing increased morbidity, mortality and hospital costs [1-3]. Selective pressure exerted by antibiotics plays a central role on the acquisition, selection, persistence and transmission of resistant pathogens which may include the following effects: (1) eradication of the susceptible skin and gut flora will increase the likelihood of acquisition of antibiotic-resistant organisms, especially in settings with endemic resistance; (2) antibiotics select for pre-existing antibiotic-resistant bacteria (ARB) in carriers and enhance the likelihood of spread; (3) antibiotic selection pressure may transform low-level carriers to high-level spreaders; (4) antibiotics active against antibiotic-susceptible bacteria may convert carriers of susceptible bacteria to non-carriers, indirectly promoting acquisition and spread of ARB; (5) antibiotic exposure may increase the risk of endogenous infection with ARB related to changes in colonisation resistance and bacterial virulence at the individual host level [4]. Numerous papers demonstrated that prior antimicrobial drug exposure is a strong risk factor for colonisation and infection due to a drug resistant pathogen [5,6]. A meta-analysis that included 76 studies for a total of 24,230 patients documented that the risk of acquiring methicillin-resistant *Staphylococcus aureus *(MRSA) was increased by 1.8-fold in patients who had taken antibiotics. Antibiotic exposure was determined in the 126 days preceding MRSA isolation [7]. Such risk was almost 3-fold greater after using quinolones or glycopeptides and 2-fold for cephalosporins. A multicentre prospective cohort study conducted in several Italian hospitals showed that 5% of patients were newly colonised by ARB including MRSA in nasal samples, after starting antibiotic therapy. Nine percent of those patients developed an infection by the same strain over the 30-day follow-up. The use of carbapenems was associated with the highest incidence of colonisation with eight new cases of MRSA colonisation for 1,000 antibiotic-day [4].

The likelihood of acquisition of nosocomial ARB during or shortly after therapy with different antibiotic agents is still unclear and is often confounded by scarce data on antibiotic usage at the individual patient level. Study design, selection bias, definition of control groups and failure to control for confounding variables might account for conflicting results [8]. No prospective multi-centre cohort study has been conducted defining, in countries with varying baseline antimicrobial resistance rates, the impact of antibiotic exposure on the development and acquisition of nosocomial carriage by ARB, after accounting for individual and group-level confounding variables, such as case-mix and colonisation pressure [8-10].

## Presentation of the hypothesis

We designed a 2-year observational cohort study (WorkPackage 4 of the EU-funded SATURN project) in order to clarify and determine the role of antibiotic therapy in the acquisition of ARB in hospitalized patients. The study will run in three European hospitals, ranging from 700 to 3,500-bed capacity: Università Cattolica Sacro Cuore "Policlinico A. Gemelli" (UCSC, Rome, Italy), Institute of Infectious Diseases "Matei Bals" (IDMB, Bucharest, Romania), and Clinical Center of Serbia (CCS, Belgrade, Serbia). Overall, 6 medical and 6 surgical wards will be included. All centers obtained ethical approval from the local institution review board.

## Testing the hypothesis

**Figure 1:**
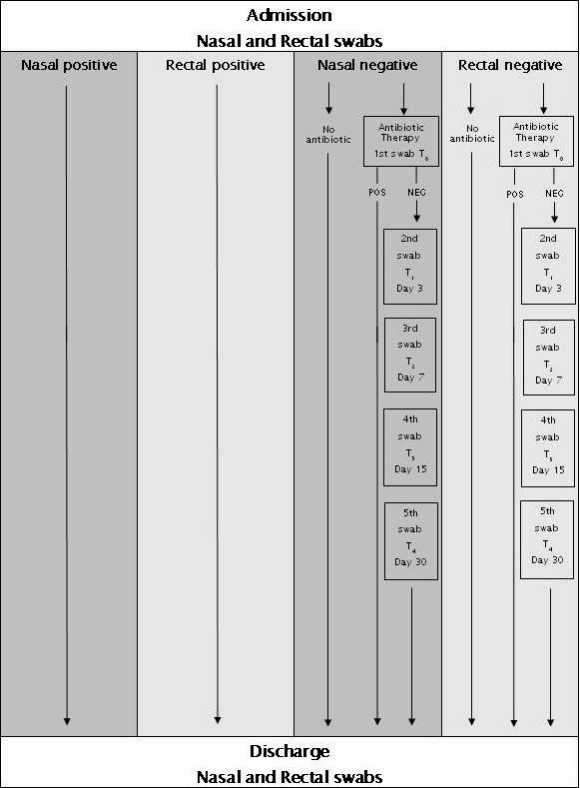
**Work flow of the SATURN WP4 clinical protocol**.

All adult patients (> 18 years) starting intravenous and/or oral antibiotic treatments during hospitalisation will be included in the study. Written informed consent will be obtained by every patient before study inclusion. Exclusion criteria will be pregnancy and recent nose surgery. Patients starting antibiotic therapy *per os *and/or intravenously will be sampled at antibiotic start (t_0_, within one hour) and at the following intervals: day 3 (t_1_), 7 (t_2_), 15 (t_3_), 30 (t_4_) (Figure 1). Patients colonised with MRSA and/or extended-spectrum β-lactamase (ESBL)-producing gram-negative bacteria before starting antibiotic therapy (t_0 _sample) will be excluded from follow-up cultures and analysis. Patients will be followed during the hospitalisation and afterwards for a total of 30-day from the inclusion in the study. Screening will be performed in outpatient clinics after patients' discharge from the hospital, if possible.

### Outcomes

The primary outcome will be the rate of acquisition of target antibiotic bacteria by 1,000 antibiotic-days in hospitalised patients. The rate will be also defined according to antibiotic class and single drug. Patients will be stratified by class of risk (e.g., cirrhosis, diabetes, malignancies) and rate of acquisition will be defined. The secondary outcome will be the rate of nosocomial infections by 1,000 days of hospitalisation in newly colonised patients after undergoing antibiotic therapy.

### Variables

The following variables will be identified in cases and controls: age; gender; reason for admission; inter and intra-wards transfers; admission from home or other acute or long-term care institutions; previous hospitalisation; surgical procedures; Intensive Care Unit (ICU) stay (previous 12 months); residency in long-term care facility; transfer form another hospital; nursing and/or medical domiciliary assistance; urinary catheter; central venous catheter (CVC, at hospital admission); underlying conditions (Infectious Disease-specific Chronic Disease Score, CDS-ID); room sharing with a ESBL or MRSA patient; previous MRSA or ESBL carrier; consciousness at admission; antibiotic therapy (previous 30 days).

Antibiotic therapy will be reported as for daily dosage, frequency of dosage(s), route of administration, type of antibiotic(s), reason for starting and stopping, duration of therapy. At every clinical follow-up the following variables will be also detected: presence of urinary catheters; CVC; surgery; room sharing with a ESBL or MRSA positive patient; MRSA or ESBL-producing gram-negative infections (including type of infection, therapy, and outcome); albumin, creatinine and blood urea nitrogen levels.

### Microbiological methods

#### Methicillin resistance detection in Staphylococcus aureus

Dedicated personnel will be trained in performing nasal samples. Both nostrils will be sampled. Specimens obtained with a dry swab (Copan, Italy) will be incubated overnight in 2 ml of Mueller-Hinton broth supplemented with colistin 32 μg/mL and 2.5% NaCl (CS) at 35°C in room air. Then 10 μl of broth sample will be plated onto chromogenic medium (MRSAid, bioMérieux, Marcy l'Étoile, France) [11] and will be incubated overnight (18-24 h) at 35°C. The growth of > 1 colonies will indicate methicillin-resistance. The isolation of *S. aureus *will be confirmed through the use of a classical DNase assay or automated phenotypic methods (VITEK2, bioMérieux, Marcy l'Étoile, France, or PhoenixTM, Becton Dickinson, Burlington, NC) [12]. Oxacillin-resistant colonies will be isolated and stored in Trypticase Soy Broth (Becton Dickinson, Burlington, NC) and glycerol 20% at -80°C in freezer vials.

#### Extended spectrum beta-lactamases detection in Enterobacteriaceae

Rectal specimens will be obtained with a dry swab (Copan, Italy) and will be inoculated onto plates of ESBL chromogenic medium (Brillaince ESBL, Oxoid, Basingstoke, UK). After the direct plating, 1 ml of trypticase soy broth (TSB) with 0.5% NaCl will be added to the transport tube and the selective broth will be incubated overnight at 37°C in air. The selective medium will be plated on the ESBL chromogenic medium following the same procedure as for direct plating. Colonies will be identified according to manufacturers' recommendations and subcultured for confirmatory testing. The ESBL phenotype of identified colonies of *Klebsiella *species, *Escherichia coli *and *Proteus *species will be detected by confirmatory double disk diffusion test (DDT). Ceftazidime (30 μg), cefotaxime (30 μg), or cefepime (30 μg) discs will be placed on Mueller-Hinton agar plates, 20 mm apart (centre to centre) from a clavulanate disc (clavulanic acid 10 μg). The test will be considered as positive when a decreased susceptibility to cefotaxime is combined with a clear-cut enhancement of the inhibition zone of cefotaxime in front of the clavulanate-containing disk, often resulting in a characteristic shape-zone referred to as 'champagne-cork' or 'keyhole' [12,13]. Plates will be incubated overnight (18-20 h) at 35°C. ESBL-producing bacteria will be stored in microbanks (Pro-Lab Diagnostic, Canada) at -80°C.

### Statistical analysis

The assessable population for each target organism will include all treated patients from whom baseline screening and at least one follow-up screening will be available. The incidence of colonisation will be defined by the number of new cases of colonisation for 1,000 days of antibiotic therapy. Only patients with a negative baseline (t_0_) will be included in the longitudinal analysis. The length of exposure to antibiotic therapy for patients newly colonised by ARB will be defined by the number of days between the inclusion in the study and the date of the first positive sample. The length of exposure to antibiotic therapy for patients not colonised will be defined by the number of days between the inclusion in the study and the date of antibiotic discontinuation. The colonisation pressure will be calculated for each center and defined as the average point of prevalence for antibiotic-resistant bacteria for all days until acquisition or discharge. Time-to-event analysis (in days) between the inclusion in the study and the acquisition of colonisation will be done using Kaplan-Meier curves and Cox proportional hazard regression modeling in which the number of days until acquisition will be the dependent variable. Two nested case-control studies will be performed:

#### Case control study 1

To define the impact of antibiotics on new acquisition of MRSA and ESBL-producing *Enterobacteriaceae*, a matched case-control study will be done (ratio 1:4). The control group will be selected among patients not receiving antibiotics, admitted in the same ward on the day of the corresponding case, with negative cultures at hospital admission. Matching criteria will include: age (± 5 years), sex, and total length of hospitalisation.

#### Case control study 2

To define individual level of risk related to specific antibiotics, patients acquiring MRSA and ESBL-producing *Enterobacteriaceae *will be compared with patients not acquiring ARB after starting antibiotic therapy (ratio 1:4). Previously known risk factors or clinically relevant significant variables from the univariate analysis will be considered for inclusion in multivariate logistic regression analysis. The Hosmer and Lemeshow goodness-of-fit test will be used to assess model fit.

### Sample size

Since the rate of acquisition of ESBL-producing gram-negative bacteria in patients receiving antibiotic therapy has not been established in prospective cohort studies, prevalence proportion was derived from retrospective case-control studies and defined as 5%. Sample size calculation for the rate of new acquisition of MRSA has been derived by a previous study where the acquisition rate, after starting antibiotic therapy, was 3% [4]. To be able to analyse 257 patients newly colonised by MRSA and 443 by ESBL-producing gram-negative, 8565 patients starting antibiotic therapy need to be enrolled. According to the number of hospital admission/year and percentage of patients starting antibiotic in each ward, the clinical centers are required to provide the following numbers of patients with new colonisation by MRSA/ESBL *Enterobacteriaceae*: 48/95, 121/201, and 88/147 for UCSC, IDMB, CCS, respectively.

## Implications of the hypothesis

SATURN project is a European multicentre study that aims to provide an in-depth knowledge of ARB acquisition through the analysis of antibiotic classes, duration of treatment and dosage in the nosocomial setting by a multidisciplinary approach. The SATURN project website http://www.saturn-project.eu provides the information and details concerning the different WPs and the entire project.

Several outcomes will be obtained at the end of the study (December 2014, expected): the rate of acquisition of target ARB by 1,000 antibiotic-days according to different classes of antibiotics, duration of therapy, and antibiotic combinations (monotherapy versus combinations); risk factors associated with new colonisation by target ARB; and risks for nosocomial infections due to target ARB after a cycle of antibiotic therapy adjusted by length of hospitalisation and ward colonisation pressure.

The WP4 study is currently recruiting patients in all clinical centers. The accomplishment of the study will be pursued thanks to the great strive of all clinicians, nurses and microbiology staff in every study step. An advertising campaign (posters, stickers and pictures) was performed in the selected wards in order to thrill and sensitise the recruited staff.

The results of the study should lead to the design of specific infection control measures for preventing spread of ARB in hospitalised non-ICU patients undergoing antibiotic therapy. A potential benefit for selective screening in high-risk patients during antibiotic therapy or shorter duration of antibiotics in such patients might be hypothesised. Readily available colonisation information could impact empiric therapy for nosocomial infections or, for example, the potential need of mupirocin prophylaxis for patients colonised by MRSA during hospitalisation. Assessment of the usefulness of the recommendations derived from the results of the SATURN study should be of valuable help for those in charge of infection control, for hospital administrators, and for those managing budgets of large healthcare organisations.

We do expect that SATURN project results will have a direct impact on clinical practice, improving the appropriateness of antibiotic prescribing policy, the prevention of antibiotic-resistant infections and the heightener of patients' quality care.

## Competing interests

The authors declare that they have no competing interests.

## Authors' contributions

GDA participated in the elaboration of clinical protocol and drafted the manuscript. GR and SV participated in the design of the study. RC reviewed the manuscript. SMK, HG, and JS elaborated the microbiological protocol. ET conceived of the study, participated in its design and coordination and helped to draft the manuscript. All authors read and approved the final manuscript.

## Pre-publication history

The pre-publication history for this paper can be accessed here:

http://www.biomedcentral.com/1471-2334/12/74/prepub
